# Targeting Cullin-RING E3 Ubiquitin Ligase 4 by Small Molecule Modulators

**DOI:** 10.33696/Signaling.2.051

**Published:** 2021

**Authors:** Kenneth Wu, Benjamin D. Hopkins, Roberto Sanchez, Robert J. DeVita, Zhen-Qiang Pan

**Affiliations:** 1 Department of Oncological Sciences, The Icahn School of Medicine at Mount Sinai, One Gustave L. Levy Place, New York, NY 10029-6574, USA; 2 Genetics and Genomics, The Icahn School of Medicine at Mount Sinai, One Gustave L. Levy Place, New York, NY 10029-6574, USA; 3 Department of Pharmacological Sciences, The Icahn School of Medicine at Mount Sinai, One Gustave L. Levy Place, New York, NY 10029-6574, USA; 4 Drug Discovery Institute, The Icahn School of Medicine at Mount Sinai, One Gustave L. Levy Place, New York, NY 10029-6574, USA

**Keywords:** E3 CRL4, Cdt1, Cell cycle, Small molecule inhibitors, Tumor inhibition

## Abstract

Cullin-RING E3 ubiquitin ligase 4 (CRL4) plays an essential role in cell cycle progression. Recent efforts using high throughput screening and follow up hit-to-lead studies have led to identification of small molecules **33-11** and **KH-4-43** that inhibit E3 CRL4’s core ligase complex and exhibit anticancer potential. This review provides: 1) an updated perspective of E3 CRL4, including structural organization, major substrate targets and role in cancer; 2) a discussion of the challenges and strategies for finding the CRL inhibitor; and 3) a summary of the properties of the identified CRL4 inhibitors as well as a perspective on their potential utility to probe CRL4 biology and act as therapeutic agents.

## Introduction

Cullin-RING (Really Interesting New Gene) E3 ubiquitin (Ub) ligases (CRLs) are RING-type E3s characterized by a signature Cullin-RING heterodimeric complex [1,2]. There are six canonical cullin (CUL) proteins, CUL1, CUL2, CUL3, CUL4A, CUL4B, and CUL5, that all adopt an elongated structure to function as a scaffold for assembly of E3 CRL. Typically, a CUL’s N-terminal domain assembles interchangeably with CUL-specific substrate receptors capable of binding a substrate. On the other hand, a CUL’s C-terminal domain (CTD) binds a RING finger protein, ROC1/RBX1 for CUL1 to 4 or ROC2 for CUL5, to form a core ligase complex. This modular feature allows for assembly of a diverse set of structurally similar, but functionally distinct CRLs. There are ~300 CRL members in humans, comprising ~50% of the E3s identified to date [1,2]. This review article focuses on E3 CRL4 and highlights recent developments aimed at targeting this E3 by small molecule inhibitors.

## E3 CRL4: Structural Organization

**Figure 1:**
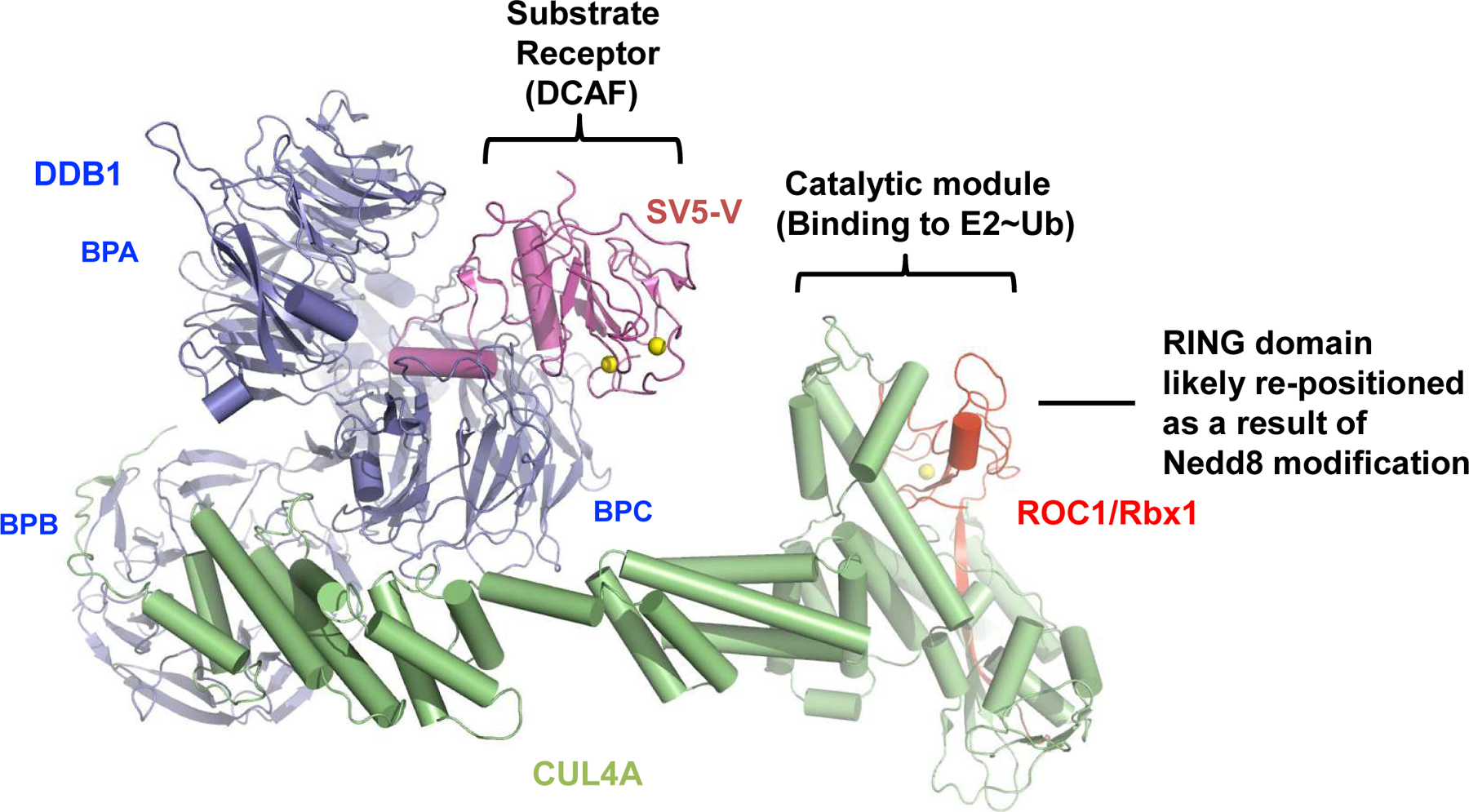
The structural organization of E3 CRL4^SV5-V^ (PDB: 2HYE).

The resolved crystal structure of CRL4A^SV5-V^ [3,4] provides an example of the overall architecture of E3 CRL4. It contains the CUL4A scaffold, the 127 kDa DNA damage-binding protein 1 (DDB1) as an adaptor, SV5-V that is a substrate receptor that belongs to the DDB1-cullin-4-associated factor (DCAF; ~90 in humans) protein family, and ROC1/RBx1 RING finger protein (Figure 1). DDB1 utilizes three β-propellers, BPA, BPB, and BPC, and a C-terminal helical domain for interactions with the N-terminal domain (NTD) of CUL4A, as well as a DCAF protein such as SV5-V, respectively. Intriguingly, comparison of a number of DDB1 structures resolved to date has revealed a significant level of plasticity, suggesting an ability of DDB1 to adopt flexible orientations that might enable E3 CRL4 to optimally position a DCAF for substrate ubiquitination, and/or accommodate substrate polyubiquitination [5].

At its C-terminus CUL4A interacts with ROC1/Rbx1 *via* both “strong” and “weak” modes. The “strong” interactions involve ROC1/RBX1’s β-strand that inserts itself between multiple CUL4A’s β-strands, creating a stable intermolecular β-sheet structure. By contrast, the rest of CUL4 C-terminal domain (CTD) appears to engage with the RING domain of ROC1/RBX1 through a loose interface. Biochemical studies using the CUL1-based CRL complexes suggest that part of the cullin-ROC1 RING domain interface interactions acts to restrain the E3-mediated ubiquitination activity and that such autoinhibition is reversed by the action of Nedd8 [6]. Nedd8 is a ubiquitin-like protein that is covalently conjugated to a conserved residue within the cullin CTD [7]. Remarkably, conjugation of CUL5 by Nedd8 was shown to cause the ROC1 RING domain to pop out [8]. This re-positioning of ROC1 RING finger likely establishes an open active conformation optimal for interactions with an E3-bound substrate and an E2~ubiquitin thiol ester, resulting in the transfer of ubiquitin to the substrate.

## The E3 CRL4^DCAF2^-Cdt1 Pathway and CRL4^CRBN^

**Figure 2:**
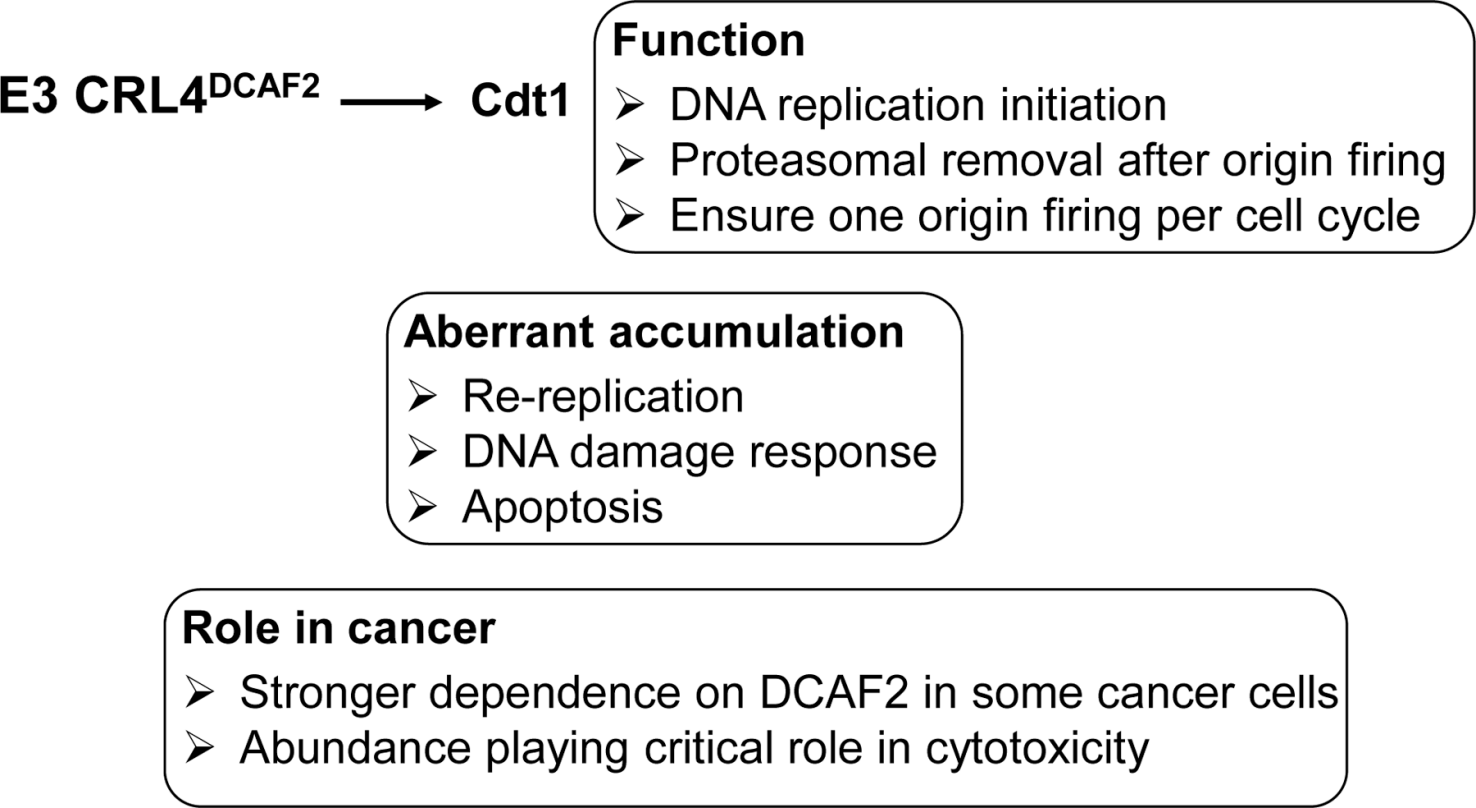
The E3 CRL4^DCAF2^-Cdt1 pathway. A list of bullet points highlights Cdt1’s function as well as physiological and pathological defects caused by aberrant accumulation of Cdt1.

Targeted degradation of Cdt1 (CDC10-dependent transcript 1) by E3 CRL4^DCAF2^ [9–11] is one of the best characterized evolutionarily conserved proteolytic mechanisms (Figure 2). Cdt1 is a DNA replication initiation factor that together with Cdc6 (cell division cycle 6), functions to load the MCM (mini-chromosome maintenance protein complex) DNA replicative helicase near to sites of replication origin. As such, Cdt1 is widely considered as a licensing factor because its action leads to replication origin unwinding and then replicative DNA synthesis [12,13]. Prompt removal of Cdt1 after origin firing by E3 CRL4^DCAF2^ and the proteasome is critical to prevent re-replication and thus ensure replication only once per cell cycle [14,15]. Of particular importance, DCAF2 (also called Cdt2) is responsible for targeting Cdt1 in a manner that requires DNA and PCNA (proliferating cell nuclear antigen). While the DCAF2/Cdt1/PCNA/DNA interactions appear complex and a detailed mechanism remains elusive, it is clear that a conserved PCNA motif known as PCNA-interacting protein (PIP) box plays a significant role [16–19].

The CRL4^DCAF2^-directed Cdt1 turnover is of fundamental importance to cell cycle progression (Figure 2). The Kipreos group first observed massive DNA re-replication with elevated DNA levels up to 100C content driven by aberrant accumulation of Cdt1 in *Caenorhabditis elegans* lacking CUL4 [14]. Similar defects have been observed in mammals. For example, mouse embryos derived from *dcaf2*-null oocytes were found to arrest at the one- to two-cell stage, owing to prolonged DNA replication and accumulation of massive DNA damage [20]. In addition, defects observed in the context of *dcaf2* deletion were phenocopied by over-expression of Cdt1 in wild type cells, supporting the notion that Cdt1 abundance is a critical determinant for cell cycle progression. As revealed by multiple studies [21–24], apoptosis is a frequently observed cellular response to DNA damage triggered by Cdt1 accumulation. The Cdt1-CRL4^DCAF2^ pathway appears to participate in DNA repair/checkpoint control. Upon exposure to UV or ionizing radiation, Cdt1 is rapidly ubiquitinated and degraded by CRL4^Cdt2^ to halt DNA replication origin firing [11,17,25–26].

CRL4^DCAF2^ has several additional substrates. It targets the degradation of cyclin-dependent kinase inhibitor p21 [27–29], and histone H4 methyltransferase Set8 [30,31]. Their reaction mechanisms may resemble that of Cdt1 because they share the same requirement for PCNA. Redundant E3s for p21 degradation have been reported. For example, the Kipreos group showed that in asynchronously growing HeLa cells, knockdown of Skp2 and DCAF2 each resulted in accumulation of p21, while the combined knockdown produced the most pronounced cooperative effect [28].

CRL4^CRBN^ has received significant attention in recent years because the DCAF protein cereblon (CRBN) was identified as a target bound to the Food and Drug Administration (FDA)-approved anticancer drug class known as Immunomodulatory imide drugs (IMiDs) that include thalidomide, lenalidomide, pomalidomide, etc [32]. Thanks to a large number of elegant genetic, biochemical and structure studies, it is now widely accepted that binding of an iMiD to CRBN creates new protein-protein interaction surface, thus enabling the iMiD-bound E3 to target a novel set of neo-substrate proteins that include IKZF1/3 (Ikaros family zinc finger protein 1/3), CK1α (casein kinase 1α), and GSPT1 (G1-to-S phase transition 1) [33–38]. Owing to the high affinity of iMiDs for CRBN, CRL4^CRBN^ has become a widely used and highly effective platform for PROTACs (proteolysis targeting chimeras), in which a dual-functional small molecule degrader can be modularly designed to contain both an iMiD and a ligand with affinity for a selected specific cellular protein targeted for degradation [39]. Once introduced into cells, the degrader is able to harness its desired targeted cellular protein substrate to E3 CRL4^CRBN^ for ubiquitination and degradation. Notably, the iMiDs-dependent ubiquitination of a neo-substrate (such as CK1α) by CRL4^CRBN^ has been reconstituted *in vitro* [36,37,40], creating tools that enable in-depth biochemical mechanistic studies and drug testing efforts. Intriguingly, a similar drug-harnessing mechanism appears to be utilized by another DCAF protein, DCAF15, that is bound to sulfonamides which then gain the ability to target RBM39 for degradation [41].

## Role of E3 CRL4 in Cancer

CUL4A and CUL4B are highly similar except that CUL4B contains a distinct N-terminal extension that includes a sequence encoding for a nuclear localization signal, thereby making it a predominantly nuclear protein [42]. CUL4A and CUL4B are broadly co-expressed. Multiple studies suggest no overt growth abnormalities in germline *Cul4a* or *Cul4b* knockout mice [43–45]. However, deletion of CUL4A and CUL4B in mouse embryonic fibroblasts, as well as cultured tumor cells, results in growth retardation [43], suggesting that the CRL4 E3 ligase activity plays an essential role in growth. Genetic ablation of the *Ddb1*, the adaptor for both CUL4A and CUL4B, is embryonic lethal [46–47]. The above findings collectively suggest that E3 CRL4 is essential for murine development, and that CRL4A and CRL4B are functionally redundant.

Previous studies have suggested the importance of both CUL4A and CUL4B in cancer [48]. Studies in mice using either overexpression or silencing approaches indicate that both CUL4A and CUL4B play a tumor-promoting role in many cancer types including lung [49,50], breast [51], colon [52,53], and hepatocellular carcinomas [54–55]. In addition, CUL4 overexpression has been linked to an array of human cancers including tumors of the breast [56], ovary [57], stomach [58–59], colon [51–53, 60], pancreas [61], lung [62] and bile duct [63–65].

Consistent with its essential role in cell cycle progression by targeting Cdt1 for degradation, deletion of the *dcaf2* gene in mice causes early embryonic lethality [66]. Elevated expression of DCAF2 is detected in ovarian cancer [21], melanoma [67], Ewing sarcoma [68], and head and neck cancer [69]. Previous studies suggest a role of the CDT1/CRL4^DCAF2^ pathway in tumorigenesis. First, it was shown that RNA interference (RNAi)-mediated depletion of DCAF2 caused apoptosis in the 12 tumor cell lines examined, but not in 6 non-transformed cell lines, suggesting that cancer cells are dependent on the functional targeting of Cdt1 by CRL4^DCAF2^ at levels much higher than normal cells [70]. Moreover, Cdt1 knockdown was able to partially reduce apoptosis in cells in which all E3 CRL activities were neutralized by the Nedd8 inhibitor MLN4924/Pevonedistat, suggesting a significant role for Cdt1 abundance in mediating genotoxic effects of the anticancer drug [21] (Figure 2).

## Targeting E3 CRL: Challenge and Strategy

A selective small-molecule modulator of CRLs’ function would facilitate mechanistic and phenotypic studies into CRL functions and provide a tool to identify their targets in biochemical, cell-based, and animal studies. To date, there is only one FDA-approved E3 drug class that targets the DCAF substrate receptor CRBN (thalidomide/lenalidomide) [71]. Current drug/probe discovery efforts against the ubiquitin-proteasome system depend heavily on traditional methods that exploit the ability of small-molecule agents to disable an enzyme’s catalytic pocket. Examples include USP7 inhibitors P50429/P22077 (modifying the de-ubiquitinating enzyme [DUB]’s catalytic cysteine residue) [72], Nedd8 inhibitor MLN4924 (forming a covalent adduct with Nedd8 to bind and inactivate Nedd8 E1 enzyme) [73,74], and proteasome drug Bortezomib (binding to, and inhibiting, the protease’s catalytic site) [75].

However, RING-type E3s are atypical enzymes that contribute to ubiquitination by mediating protein–protein interactions (PPI) with substrate, E2, and Ub [1]. Previous successful PPI drugs typically exploit a deep hydrophobic pocket in a target. For example, E3 MDM2 (mouse double minute 2) has a deep hydrophobic cavity formed by 14 amino acids, with which three amino acids from p53 directly interact [76]. Several small molecule inhibitors including nutlins [77; reviewed by ref. 78], have been developed and their mechanism of action is to occupy the MDM2 pocket and displace p53, thereby protecting p53 from being degraded by MDM2. However, high-resolution structural studies have shown that interactions involving E3’s RING domain, E2, and Ub are characterized by large, relatively flat interfaces [79]. Such perceived “undruggable” features impose a significant barrier to utilize structure-based ligand search using either virtual docking or fragment based physical screening for the discovery of CRL RING domain PPI drugs.

**Figure 3:**
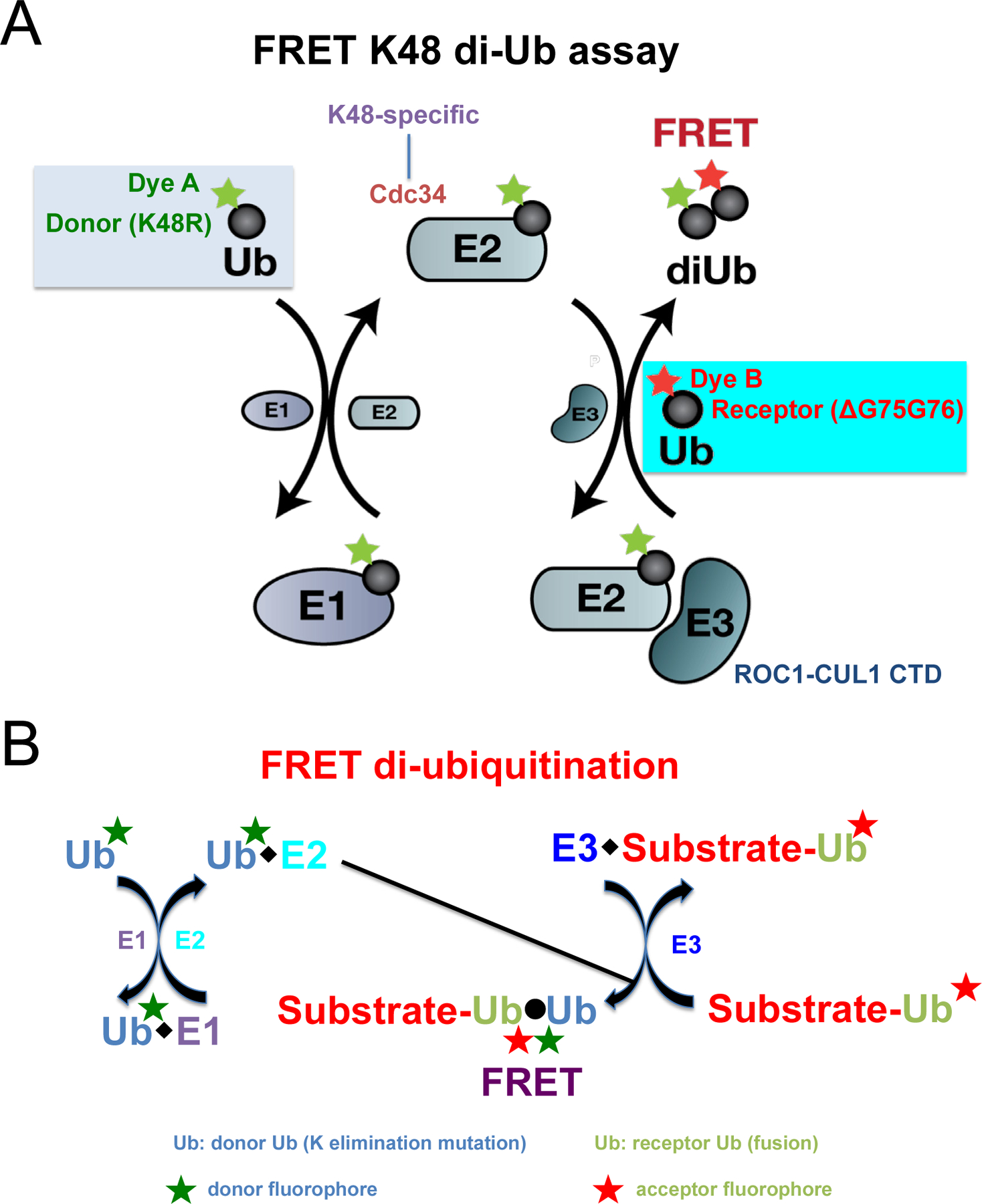
FRET strategies to target E3 CRLs. **A)** FRET K48 di-Ub assay scheme. It begins with formation of a thiolester complex by E1 and Ub-Q31C/K48R-iFluo 555. This is followed by transthiolation to produce E2 Cdc34~Ub-Q31C/K48R-iFluo 555. In the presence of E3 sub-complex ROC1-CUL1 CTD, the K48 residue of Ub-E64C/ΔGG-iFluo 647 attacks Cdc34~Ub-Q31C/K48R-iFluo 555, yielding a di-Ub covalent product. As a result, Ub-linked fluorophores iFluo 555 and iFluo 647 are brought into proximity that generates FRET upon donor fluorophore excitation. **B)** FRET di-ubiquitination scheme. The final outcome is a result of mixing two reaction assemblies. Assembly 1 (*right hand*) is a complex formed by an E3 and a substrate-Ub fusion molecule. Ub is labeled with red coded acceptor fluorophore. Assembly 2 (*left hand*) involves reactions of the donor Ub with E1 and E2 to form E2~donor Ub thiol ester complex. The donor Ub contains a lysine substitution that eliminates Ub chain assembly and is labeled with blue coded donor fluorophore. The combination of assemblies 1 and 2 produces a uniform substrate-di-Ub product, resulting in close proximity between the red and blue fluorophores. When the donor fluorophore is excited, its emission causes the excitation of the acceptor fluorophore, which emits a distinct fluorescent signal.

To address the need to find a chemical probe for E3 CRL’s core ligase complex, a high-throughput screening (HTS) platform was created using the fluorescence (Förster) resonance energy transfer (FRET) K48 di-Ub assay (80–81) (Figure 3A). In this system, incubation of E1, E2 Cdc34, and an E3 CRL1 subcomplex (ROC1-CUL1 CTD) results in covalent conjugation of the donor Ub to the receptor Ub. Because the donor and receptor ubiquitins carry a pair of matching fluorophores at specific sites, the conjugation of these two molecules allows for optimal FRET energy transfer from the excited donor fluorophore to the receptor fluorophore, producing a distinct fluorescence signal. Fully functional Ub variants were employed to allow only one nucleophilic attack. As a consequence, a single Ub-Ub isopeptide bond is produced, thereby eliminating the complexity associated with polyubiquitin chain assembly which ensures a high degree of reproducibility for effective HTS. A pilot HTS identified a small molecule compound, suramin (an anti-trypanosomal drug), that can inhibit E3 CRL1 activity by disrupting its ability to recruit E2 Cdc34 [80]. These results have provided proof-of-principle evidence that an E2–E3 interface can be perturbed with small molecule modulators. Using this approach, large-scale HTS with extensive follow-up hit-to-lead studies, led to identification of a class of small-molecule inhibitors against E3 CRL4 [82].

More recently, a FRET diubiquitination assay (Figure 3B) was developed to track substrate ubiquitination by fluorescence [83]. While FRET diubiquitination is derived from the FRET K48 di-Ub assay (Figure 3A), it requires E3-substrate interactions. Hence this new reporter system could be used in the future HTS campaigns to discover small-molecule modulators capable of targeting specific substrate-E3 interfaces.

## Discovery of Inhibitors against E3 CRL4

**Figure 4:**
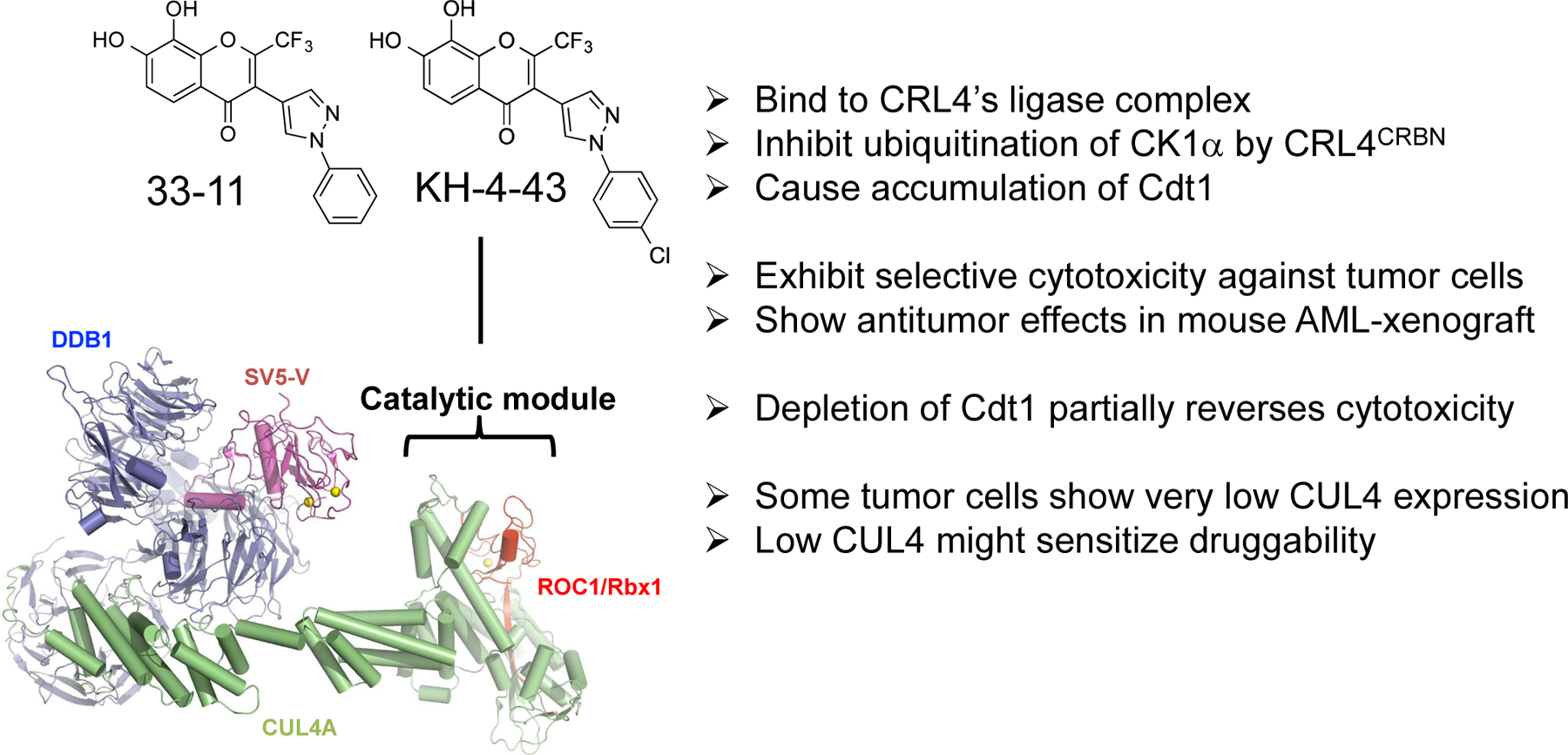
**33-11**/**KH-4-43**: inhibitors of E3 CRL4 with anticancer potential. Newly identified CRL4 inhibitors **33-11** and **KH-4-43** target E3’s core ligase module. Highlighted are the properties of the inhibitors.

In an effort to discover small molecule modulators that target E3 CRL, HTS and follow up hit-to-lead studies were carried out, resulting in identification of two compounds, **33-11** and **KH-4-43**, which inhibit E3 CRL4 and exhibit antitumor potential [82]. These compounds bind to CRL4’s core catalytic complex, inhibit E3 CRL4A^CRBN^-dependent ubiquitination of CK1α *in vitro*, and cause stabilization of CRL4’s substrate Cdt1 in cells (Figure 4). Treatment with **33-11** or **KH-4-43** in a panel of 36 tumor cell lines revealed cytotoxicity, which was linked to aberrant accumulation of Cdt1 known to trigger apoptosis.

According to the Structural Genomics Consortium, chemical probes are required to minimally have *in vitro* potency of the target protein at <100 nM, possess >30X selectivity relative to other sequence-related proteins of the same target family, and have demonstrated on-target effects at <1 μM [84]. **KH-4-43** appears close to these criteria because it has a binding K_d_ to E3 ROC1-CUL4A CTD or the highly related ROC1-CUL1 CTD at 83 nM or 9.4 μM, respectively [82], which represents a difference of two orders of magnitude. **KH-4-43** exhibits cytotoxicity in a subset of tumor cell lines with EC_50_ approaching ~2 μM. The on-target effects were supported by the results of RNAi experiments, which demonstrated that **33-11**’s cytotoxicity was enhanced by CUL4 depletion, but was partially overcome by knockdown of CDT1.

Comparison of **KH-4-43** and **33-11** revealed improved potency for **KH-4-43** as an inhibitor of E3 CRL4 [82]. **KH-4-43** is more effective than **33-11** in binding to CRL4’s core ligase subcomplex, inhibiting the ubiquitination of CK1α by E3 CRL4^CRBN^ *in vitro*, and stabilizing CRL4’s substrate CDT1 in cells. **KH-4-43** exhibits higher levels of cytotoxicity and *in vivo* antitumor activity than **33-11**.

Thus, it appears possible for a small molecule agent to selectively target a specific cullin CTD. Despite a common globular CTD adopted by cullins 1 to 5, however, different cullin CTDs have divergent folds [4,8,85,86] and different total areas of interface with ROC1/Rbx1 that result in significantly divergent orientation of the ROC1/Rbx1 RING domain among CRLs [86]. It is therefore conceivable that a small molecule (such as **33-11**/**KH-4-43**) may bind to a specific site within a cullin CTD that impacts the cullin-E2 interaction and/or alters the ROC1/Rbx1 orientation, leading to selective inhibition of ubiquitination.

The **33-11**/**KH-4-43** class of inhibitors may prove useful as tools for probing CRL-mediated biological signaling pathways and mechanisms. A co-crystal structure of E3 CRL4 with **33-11**/**KH-4-43** (or improved analog) will reveal critical insights into the ligand-target interactions to guide structure-based design of compounds to improve inhibitory potency and selectivity. Moreover, by determining the binding mode of **33-11**/**KH-4-43** on CRL4, such studies may reveal how ligand-E3 interactions might inhibit ubiquitination, hence providing fresh insights for the use of chemical biology strategies to modulate CRL activity in a manner that broadly impacts the ubiquitin-proteasome field. Proteomic approaches using **33-11**/**KH-4-43** may identify cellular targets that discover new E3-mediated biological interactions and pathways. For example, the Ebert group has employed a SILAC-based global search of protein abundance changes in cells treated with lenalidomide, which helped identify substrate CK1α [36].

Based on preliminary structure activity relationship (SAR) studies, the **33-11**/**KH-4-43** scaffolds are highly amenable to medicinal chemistry optimization with several substituents available for modification and building blocks that have been easily incorporated into the scaffold [82]. Continued characterization of the key pharmacophores of validated leads will provide improved CRL4 potency and selectivity, as well as drug-like properties and pharmacokinetic parameters.

## Inhibitors of E3 CRL4 Have Anticancer Potential

Inhibitors of E3 CRL4 **33-11**/**KH-4-43** are toxic to a panel of tumor cells and such cytotoxicity can be partially reversed by depletion of Cdt1 [82]. These findings suggest that aberrant accumulation of CDT1, as a result of inactivation of E3 CRL4 by **33-11**/**KH-4-43**, triggers apoptosis in some cancer cells. Intriguingly, a subset of tumor cells was found to express CUL4 proteins at levels as much as 70-fold lower than those in other tumor lines [82]. In a strong support of the key role played by CUL4 abundance in these drug effects, it was shown that reducing CUL4 levels by means of siRNA-mediated depletion sensitized U2OS cells to **33-11** treatment for apoptosis [82]. **33-11**/**KH-4-43** exhibited *in vivo* anti-tumor activity in an Acute Myelogenous Leukemia (AML) xenograft mouse model study [82]. Taking together, these findings suggest that **33-11**/**KH-4-43**-based CRL4 inhibitors may provide new exploitable therapeutic opportunities to target against a subset of leukemias that are characterized by low CUL4 expression (Figure 4).

The major impact of low target expression in enhancing druggability has been previously recognized as clinically significant. A well-known example, leukemic del(5q) myelodysplastic syndrome cells, are haplo-insufficient for CK1α and these cells are sensitized to lenalidomide therapy that specifically targets CK1α for degradation [36]. If validated, the low-target expression driven drug sensitivity mechanism may be advantageous as compared with targeting amplified or mutated oncoproteins because targeting these oncogenic drivers frequently leads to selection events such as secondary mutations which result in drug resistance [87].

## Concluding Remarks

Targeting the ubiquitin-proteasome system is a new frontier for drug discovery [71]. E3 CRLs play a critical role in protein homeostasis and cell maintenance. Their functions are critical for tumor cell survival making them potentially actionable targets for cancer therapy. Through the development of assays and tools that allow us to interrogate the function and requirements for specific CRLs in normal and tumor cells we can gain insight into tumor specific dependencies upon specific CRLs and develop agents that can specifically modulate their activities.

An overwhelming amount of data demonstrates that the abundance of Cdt1 is a critical determinant for cell cycle progression. Dysregulation of the Cdt1/CRL4^DCAF2^ pathway causes re-replication and cellular DNA damage response that includes apoptosis. Cdt1 has become a significant biomarker that mediates the genotoxic effects of anticancer drugs such as MLN4924/Pevonedistat [21] and recently identified CRL4 inhibitor **33-11** [82]. However, the cytotoxicity of MLN4924/Pevonedistat and **33-11** can be rescued only partially by the depletion of Cdt1 [21,82], suggesting contributions by additional factors. Broader investigation of substrate spectrum and biological signaling pathways affected by CRL4 inhibition may lead to identification of previously unknown mechanisms in cancer biology and therapeutics.
